# Anti-Obesity Effects of Spiramycin *In Vitro* and *In Vivo*

**DOI:** 10.1371/journal.pone.0158632

**Published:** 2016-07-11

**Authors:** Mun Ock Kim, Hyung Won Ryu, Ji-Hee Choi, Tae Hyun Son, Sei-Ryang Oh, Hyun-Sun Lee, Heung Joo Yuk, Sungchan Cho, Jong Soon Kang, Chang Woo Lee, Jinhyuk Lee, Chong-Kil Lee, Sung-Tae Hong, Su Ui Lee

**Affiliations:** 1 Natural Medicine Research Center, Korea Research Institute of Bioscience and Biotechnology, 30 Yeongudanji-ro, Ochang, Cheongju, Chungbuk, 28116, Korea; 2 Anticancer Agent Research Center, Korea Research Institute of Bioscience and Biotechnology, 30 Yeongudanji-ro, Ochang, Cheongju, Chungbuk, 28116, Korea; 3 Bio-Evaluation Center, Korea Research Institute of Bioscience and Biotechnology, 30 Yeongudanji-ro, Ochang, Cheongju, Chungbuk, 28116, Korea; 4 Korean Bioinformation Center (KOBIC), Korea Research Institute of Bioscience and Biotechnology, 125 Gwahak-ro, Yuseong, Daejeon, 34141, Korea; 5 College of Pharmacy, Chungbuk National University, Cheongju 28644, Korea; 6 Department of Biological Sciences, Korea Advanced Institute of Science & Technology, 291 Daehak-ro, Yuseong, Daejeon, 34141, Korea; East Tennessee State University, UNITED STATES

## Abstract

The effects of spiramycin on adipogenesis and high fat diet (HFD)-induced obesity were investigated. Potential mechanisms contributing to these effects were elucidated. The inhibitory effect of spiramycin on adipocyte differentiation was assessed using 3T3-L1 preadipocyte cells, in which several parameters involved in AMPK signal pathways and lipid metabolism were examined. To further investigate the pharmacological effects of spiramycin *in vivo*, we examined several obesity-related parameters in HFD-induced obese mice. Spiramycin significantly inhibited preadipocyte differentiation by attenuating intracellular lipid accumulation. Spiramycin also reduced the expression of adipogenic master regulators (PPARγ, C/EBPα, and SREBP1c) and their downstream target genes (FAS, aP2, and GLUT4) in 3T3-L1 cells. In addition, AMPK phosphorylation was increased by spiramycin treatment in 3T3-L1 cells during early differentiation. Notably, HFD-induced obese mice administered spiramycin showed substantial decreases in body weight gain, serum leptin levels, adipose tissue mass, and hepatic lipid accumulation. Moreover, the decreased levels of GPT and GOT in the serum indicated that spiramycin attenuated hepatic injury caused by HFD. Taken together, these results demonstrate for the first time that spiramycin effectively attenuates HFD-induced obesity and hepatic steatosis by inhibiting adipogenesis.

## Introduction

Obesity is associated with serious human diseases, including type 2 diabetes, hyperlipidemia and cancer [1]. Obesity is characterized by increases in the number and size of adipocytes in adipose tissue [2]. Adipocytes store excess energy in the form of triglycerides and release energy as glycerol and fatty acids [3]; therefore, altered adipocyte function is associated with metabolic disorders. Numerous studies suggest that some metabolic disorders might be treated or prevented by inhibiting adipogenesis and regulating adipocyte function, although the molecular mechanisms underlying such effects remain unclear [4, 5].

Adipogenesis is accompanied by morphological and biochemical changes in adipose tissue [6]. Preadipocytes undergo growth arrest, clonal expansion, and terminal differentiation to become mature adipocytes. Upon hormonal induction, multiple transcription factors involved in inducing adipogenesis are sequentially activated [7, 8]. During this early stage of adipogenesis, CCAAT/enhancer-binding protein (C/EBP)β and δ induce expression of peroxisome proliferator-activated receptor (PPAR) γ, which induces C/EBPα, which plays a critical role in sterol regulatory element-binding protein (SREBP) 1c expression during the later stages of adipogenesis. After differentiation, adipocytes control lipid metabolism through lipogenic proteins (e.g. fatty acid synthase (FAS) and acetyl-CoA carboxylase (ACC)) and lipolytic enzymes (e.g. hormone-sensitive lipase).

AMP-activated protein kinase (AMPK) monitors intracellular energy status and regulates fatty acid-uptake and metabolism [9]. AMPK stimulates energy-producing processes such as fatty acid oxidation, glycolysis and ketogenesis. Moreover, AMPK inhibits energy-consuming processes such as lipogenesis, protein synthesis, and gluconeogenesis [10]. In adipocytes, AMPK phosphorylates several metabolic proteins, including acetyl-CoA carboxylase (ACC), thereby inactivating ATP-consuming biosynthetic processes, including lipid synthesis. Also, AMPK regulates PPARγ and C/EBPα, which are the central regulators of adipogenesis and lipid storage in adipocytes [11]. Indeed, AMPK activators such as resveratrol and EGCG (the most abundant catechin in tea) inhibit adipogenesis *in vitro* and prevent high-fat diet (HFD)-induced obesity *in vivo* [12–16]. Therefore, activated AMPK signaling has an important role in preventing obesity (or fat accumulation) and related metabolic diseases [10].

There is a demand for safe and potent anti-obesity drugs, because currently available anti-obesity drugs, such asorlistat (gastrointestinal lipase inhibitor) and fibrates (PPARα agonists), cause undesirable side effects. Therefore, we screened a natural compound library and identified spiramycin as a hit molecule showing anti-adipogenic activity. In the present study, we show that spiramycin inhibits adipogenesis in 3T3-L1 cells and ameliorates obesity and associated metabolic indications in HFD-fed mice.

## Materials and Methods

### Chemicals and reagents

Spiramycin (S-9132), insulin, 3-isobutylmethylxanthine (IBMX), dexamethasone, orlistat, and Oil Red O were purchased from Sigma-Aldrich (St. Louis, MO, USA). Dulbecco's modified Eagle's medium (DMEM), fetal bovine serum (FBS), phosphate-buffered saline (PBS), penicillin-streptomycin, and calf serum were purchased from Gibco BRL (Grand Island, NY, USA). SYBR Green reaction buffer was purchased from Bio-Rad (Hercules, CA, USA). Anti-PPARγ, anti-C/EBPα, anti-SREBP1, and anti-α-tubulin antibodies were purchased from Santa Cruz Biotechnology (Santa Cruz, CA, USA). Anti-AMPKα, anti-ACC, anti-phospho-AMPKα (Thr172), and anti-phospho-ACC (Ser79) antibodies were obtained from Cell Signaling Technology (Beverly, MA, USA).

### Cell culture and adipocyte differentiation

3T3-L1 mouse embryonic preadipocyte cells were purchased from the American Type Culture Collection (CL**-**173; ATCC, Manassas, VA, USA). 3T3-L1 preadipocytes were passaged in growth medium (GM; high glucose DMEM supplemented with 10% calf serum, 100 U/mL of penicillin, and 100 μg/mL of streptomycin), with a change of medium every 2 days, in a humidified atmosphere of 5% CO_2_ at 37°C. To induce adipocyte differentiation (S1 Fig), 3T3-L1 preadipocytes were cultured until post-confluence (Day 0), at which point the GM was replaced with fresh differentiation medium (DM; high glucose DMEM with 10% FBS including MDI (isobutylmethylxanthine (IBMX, 0.5 mM), dexamethasone (1 μM), and insulin (1 μg/mL))) for 2 days in the presence of spiramycin. On day 2 (Day 2), the medium was replaced with DM (high glucose DMEM with 10% FBS containing 10 μg/ml insulin only). After 2 days (Day 4), the cells were re-fed with fresh high-glucose DMEM with 10% FBS without MDI. On day 6 (Day 6), fully differentiated cells were analyzed for each experiment. A schematic representation of the procedure is presented in S1A Fig.

### Oil red O staining

On 6 days after the differentiation (Day 6), fully differentiated 3T3-L1 adipocytes were fixed for 1 h at room temperature with 10% formalin in PBS, washed three times with PBS, and then stained for 1 h with filtered Oil Red O (0.5% in 60% isopropanol). After the cells were washed three times with distilled water, the stained cells were photographed under a microscope. Intracellular Oil Red O was extracted with 100% isopropanol. The absorbance at 520 nm was measured using an Epoch spectrophotometer (Biotek, Winooski**,** VT**,** USA).

### Screening of natural compounds inhibiting adipogenesis

Spiramycin was identified from a natural compound library in which each compound was isolated from microbial source and medicinal plants used in traditional medical practices. The selected natural compounds were dissolved in dimethyl sulfoxide (DMSO, Sigma-Aldrich). 3T3-L1 preadipocyte cells (8×10^3^/well) were seeded in 96-well plates and differentiated in DM containing each selected natural compound for 2 days. After induction of adipocyte differentiation, cellular lipid accumulation was measured using Oil Red O.

### Triglyceride quantification assay

Cellular triglyceride content was determined using a Triglyceride Quantification Kit (BioVision, Mountain View, CA) according to the manufacturer's instructions. Briefly, the cells were rinsed three times with PBS, extracted with lipid extraction solution, and slowly heated to 90°C for 30 min. Insoluble cellular debris was removed by centrifugation for 10 min at room temperature. The triglyceride concentration in the supernatant was determined by an enzyme-based colorimetric assay. The absorbance at 570 nm was measured using an Epoch spectrophotometer (Biotek, Winooski**,** VT**,** USA).

### Evaluation of mRNA expression levels

Total RNA was isolated with TRIzol reagent (Invitrogen, Carlsbad, CA, USA) according to the manufacturer’s protocol. The concentration and purity of total RNA were calculated using a NanoDrop Spectrophotometer at 260 and 280 nm (Thermo Fisher Scientific, Waltham, MA, USA). The first cDNA strand was synthesized with 2 μg of total RNA and 1 μM of Oligo-dT_18_ primer using Omniscript Reverse Transcriptase (Qiagen, Valencia, CA, USA). Quantitative real-time PCR (qRT-PCR) amplification was performed using an S1000 thermal cycler real-time PCR system and iQ SYBR Green supermix (Bio-Rad, Hercules, CA, USA) in the presence of first-strand cDNA (1:25 dilution) and 20 pmol of primers according to the manufacturer’s protocols. The primers used to amplify specific products are listed in Table 1. All reactions were run in triplicate and data were analyzed by the 2^−ΔΔCT^ method. Significance was determined by a two-tailed Student's *t*-test (*p<0.05, **p<0.01, and ***p< 0.001).

**Table 1:** Primer sequences used in this study.

Target gene	Forward primer (5’-3’)	Reverse primer (5’-3’)
*PPARr*	CCCTGGCAAAGCATTTGTAT	GAAACTGGCACCCTTGAAAA
*C/EBPα*	GCAAAGCCAAGAAGTCGGTG	AGGCGGTCATTGTCACTGGT
*SREBP1c*	TGTGGCAGTGGAGGAGGCACA	CCGCTGGGCTTTGACCTGGC
*aP2*	CATCAGCGTAAATGGGGATT	TCGACTTTCCATCCCACTTC
*GLUT4*	CTCCTTCTATTTGCCGTCCTC	CTGTTTTGCCCCTCAGTCATT
*FAS*	ACATGGTAGCTGCCCTCAAG	GCGCAGTACCGTAGAAGGAC
*β-actin*	AGGCTGTGCTGTCCCTGTATGC	ACCCAAGAAGGAAGGCTGGAAA

### Western blot analysis

The cultured cells were washed with ice-cold PBS, harvested using a cell scraper, and lysed in ice-cold buffer (50 mM Tris-HCl (pH 8.0), 5 mM EDTA, 150 mM NaCl, 1% NP-40, 0.1% SDS, 1 mM PMSF, and 1×protease inhibitor cocktail (Roche, Penzberg, Germany)). The cell lysates were centrifuged to remove cell debris. The protein concentrations of the lysates were measured using protein assay reagent (Bio-Rad Laboratories, Hercules, CA, USA). The cell lysates were resolved by electrophoresis on 10–12% polyacrylamide gels containing sodium dodecyl sulfate and transferred to polyvinylidene difluoride membranes. The membranes were blocked in Tris-buffered saline containing 5% nonfat dry milk and 0.1% Tween-20 for 1 h at room temperature. After incubation overnight at 4°C with primary antibodies, the membranes were incubated with horseradish peroxidase-conjugated secondary antibodies for 1 h at room temperature. Protein bands were visualized using a LAS-4000 luminescent image analyzer and Multi Gauge software, version 3.0 (Fujifilm, Tokyo, Japan).

### Animal care and treatment

The experimental protocols were approved by the Institutional Animal Care and Use Committee of the Korea Research Institute of Bioscience and Biotechnology. 40 male C57BL/6 mice (6 weeks old) were housed in a controlled atmosphere (25 ± 1°C at 50% relative humidity) with a 12-h light/dark cycle. The mice were housed with 8 mice per cage and given water *ad libitum*. After acclimation for 1 week, mice were randomly assigned to 1 of 5 groups with equal mean body weight between groups, and fed specific diets as follows: standard chow diet (control, one group, 20% protein, 70% carbohydrate, 10% fat, by Research Diets Inc. #D12450B) or HFD (four groups, 20% protein, 20% carbohydrate, 60% fat, by Research Diets Inc. #D12492). After 4 weeks, when the HFD-fed mice were significantly obese in comparison with the normal diet-fed mice, the treatment regimen was started. Either spiramycin or orlistat (positive control), dissolved in 0.5% carboxy methyl cellulose (CMC), was administered 5 times per week by oral gavage for 8 weeks. Mice in the vehicle control group were given an equal volume of 0.5% CMC by oral gavage. Food intake and body weight were measured 3 times per week. At the end of the experiment, all animals were fasted overnight and sacrificed by CO_2_ asphyxiation. Blood samples were collected from the posterior vena cava and plasma was prepared for biochemical analysis and leptin ELISA. Subcutaneous fat, epididymal fat, and mesenteric fat, and the liver were removed surgically, weighed, and immediately frozen in liquid nitrogen.

### Biochemical analysis

Levels of triglycerides, total cholesterol, high-density lipoprotein(HDL) cholesterol, low-density lipoprotein (LDL) cholesterol, glutamic-oxaloacetic transaminase (GOT), and glutamic-pyruvic transaminase (GPT) in plasma were measured using a chemical analyzer (AU400, Olympus, Tokyo, Japan). Plasma leptin levels were assayed using a mouse leptin ELISA Kit (R&D Systems, Inc., Minneapolis, MN, USA) according to the manufacturer’s instructions.

### Histological staining

Sections of epididymal adipose tissue and liver were fixed in 10% formalin, dehydrated, and embedded in paraffin. Adipose tissue and liver sections were stained with hematoxylin and eosin (H&E) to allow morphological examination.

### Statistical analysis

Data are presented as mean ± standard deviation (SD). Statistical analysis was performed using Student's *t*-test for the *in vitro* experiments. Differences were considered significant at *p*<0.05 (*), *p*< 0.01 (**), and *p*< 0.001 (***). Two-way ANOVA followed by Bonferroni’s multiple comparison test was used for the analysis of body weight and food intake. One-way ANOVA followed by Dunnett’s multiple comparison test was used for fat mass, liver weight, leptin levels, GOT, and GPT analyses. A value of *p* < 0.05 was considered statistically significant.

## Results

### Spiramycin inhibits lipid accumulation during adipogenesis

**Fig 1:**
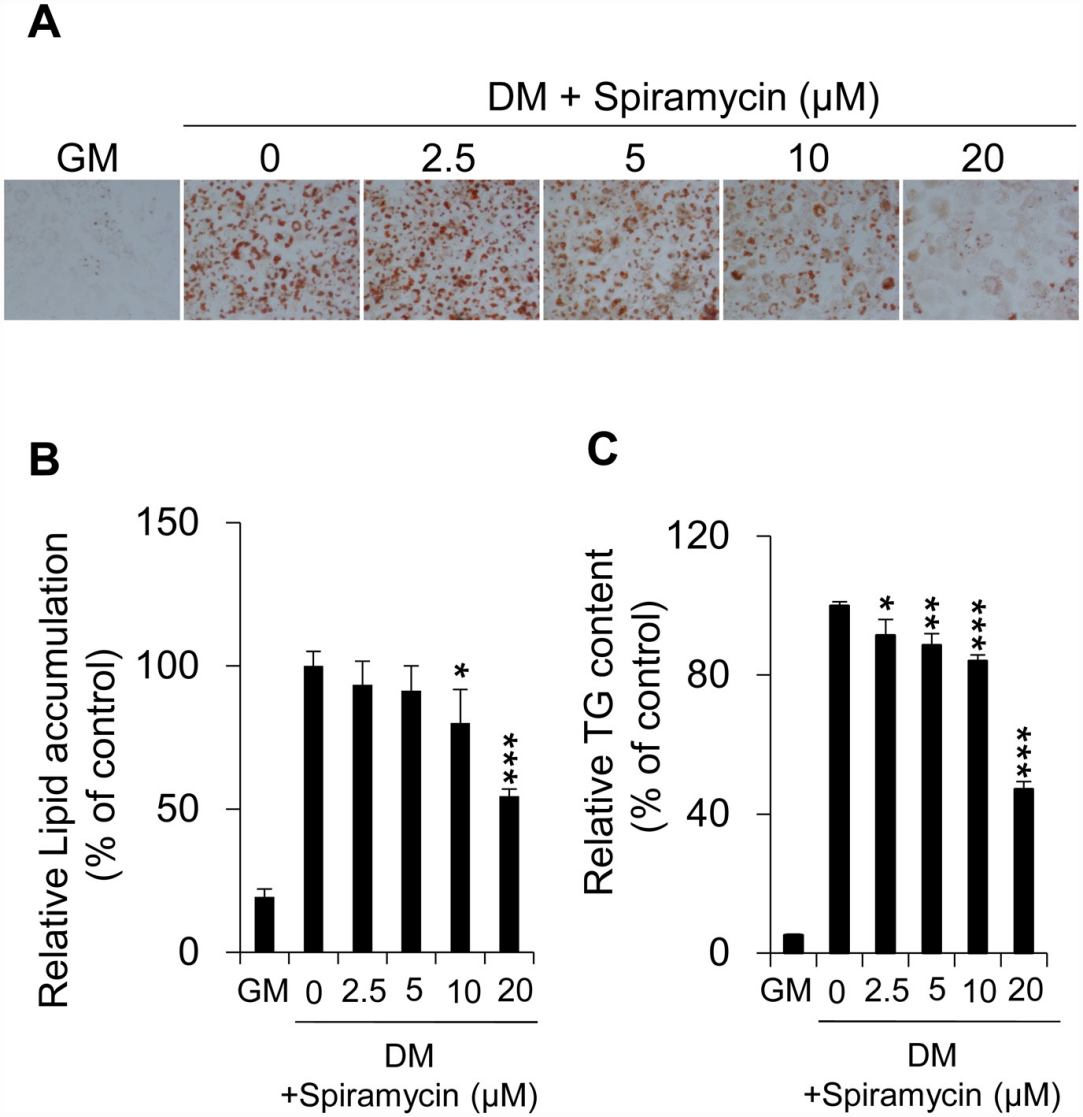
Inhibitory effect of spiramycin on lipid accumulation in 3T3-L1 adipocytes. (A) The effect of spiramycin on lipid droplet formation was measured by Oil Red O staining. 3T3-L1 preadipocytes were differentiated into adipocytes in the presence of various concentrations of spiramycin (2.5–20 μM) for 6 days. Representative cell images were captured at 200× magnification. GM, growth media. DM, differentiation media. (B) Quantification of intracellular lipid accumulation. Oil Red O stained lipids were extracted in absolute isopropanol, after which the absorbance of the solution was measured at 510 nm. (C) Quantification of triglyceride content. The bar graphs show the mean ± S.D. of 3 independent experiments (*p< 0.05, **p< 0.01, and ***p< 0.001 compared with the non-spiramycin-treated DM control).

To investigate the effect of spiramycin on adipogenesis in 3T3-L1 preadipocytes, post-confluent 3T3-L1 cells were induced by differentiation medium (DM) including MDI in the presence of spiramycin. On day 6 after differentiation (Day 6; S1A Fig), spiramycin-treated preadipocytes showed a dose-dependent reduction in Oil red O-stained lipid droplet formation in comparison with that of non-spiramycin-treated positive control cells (Fig 1A, 0 vs. 2.5–20 μM spiramycin). Spiramycin showed no cytotoxicity in 3T3-L1 preadipocytes during adipocyte differentiation (S1B Fig). Quantitative analysis of Oil red O-stained lipids in spiramycin-treated preadipocytes showed that spiramycin significantly reduced lipid accumulation to approximately 60% of the non-spiramycin-treated control cells (Fig 1B). Consistent with these results, intracellular triglyceride accumulation in spiramycin-treated cells was significantly decreased in a dose-dependent manner in comparison with that of non-spiramycin-treated control cells (Fig 1C). The results demonstrate that spiramycin inhibits adipogenesis in 3T3-L1 preadipocytes, thereby reducing lipid accumulation.

### Spiramycin decrease adipogenic gene expression by reducing expression of PPARγ, SREBP1c, and C/EBPα

**Fig 2:**
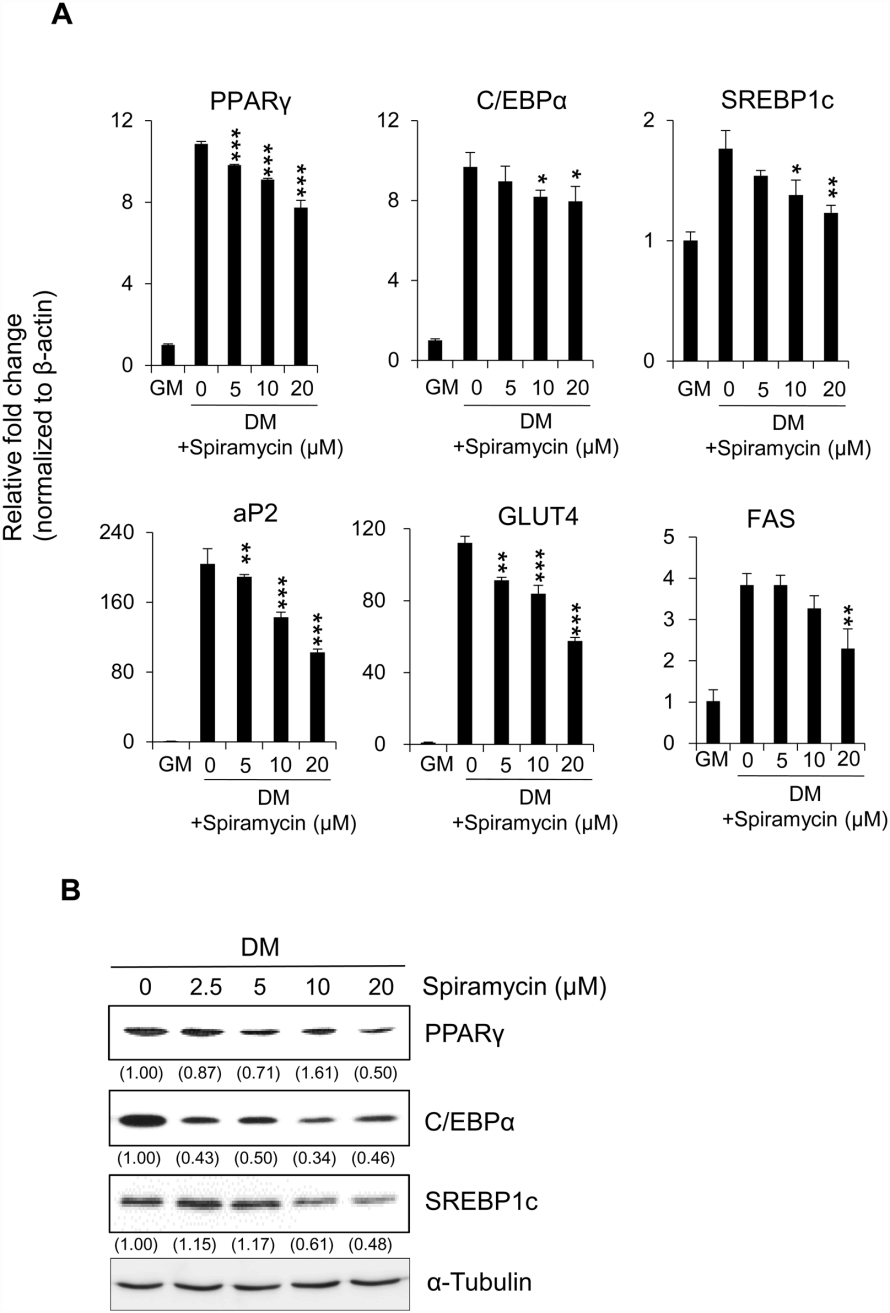
Inhibitory effect of spiramycin on expression of adipogenic transcriptional factors and their adipocyte-specific target genes in 3T3-L1 adipocytes. (A) 3T3-L1 preadipocytes were differentiated into adipocytes in the presence of spiramycin for 6 days. The transcript levels of major adipogenic transcription factors (PPARγ, C/EBPα, and SREBP1) and their adipocyte-specific target genes (aP2, GLUT4, and FAS) were evaluated by qRT-PCR. (B) Western blotting analysis showing the effect of spiramycin on protein levels of major adipogenic transcription factors (PPARγ, C/EBPα, and SREBP1). The numbers at the bottom of the figure indicate the relative intensity of each band (fold-change in comparison with that of the control group), which was estimated using Multi Gauge software version 3.0.

Adipocyte differentiation requires expression of adipogenic genes regulated by adipogenic transcriptional factors, such as PPARγ, C/EBPα, and SREBP1c. To verify the mechanism underlying attenuation of adipogenesis by spiramycin, we checked the gene regulation for adipogenesis in spiramycin-treated preadipocytes using qRT-PCR and western blotting analysis. Notably, spiramycin considerably reduced transcriptional expression of master transcriptional factors for adipogenesis, including PPARγ, C/EBPα, and SREBP1c, as well that of their target genes (*FAS*, *aP2*, and *GLUT4*), in 3T3-L1 cells (Fig 2A). Consistent with these results, protein levels of adipogenic transcription factors PPARγ, C/EBPα, and SREBP1c were also reduced by spiramycin treatment (Fig 2B). These results show that spiramycin effectively inhibits adipogenesis by attenuating expression of key adipogenic transcription factors and their adipogenic target genes.

### Spiramycin increases AMPK activity during early adipogenesis

AMPK is an important regulator of adipogenesis and lipogenesis. Activated AMPK by phosphorylation at Threonine-172 of AMPKα subunit phosphorylates various lipid metabolism-associated substrates, including ACC, thereby inhibiting adipogenesis and fatty acid formation [17].

**Fig 3:**
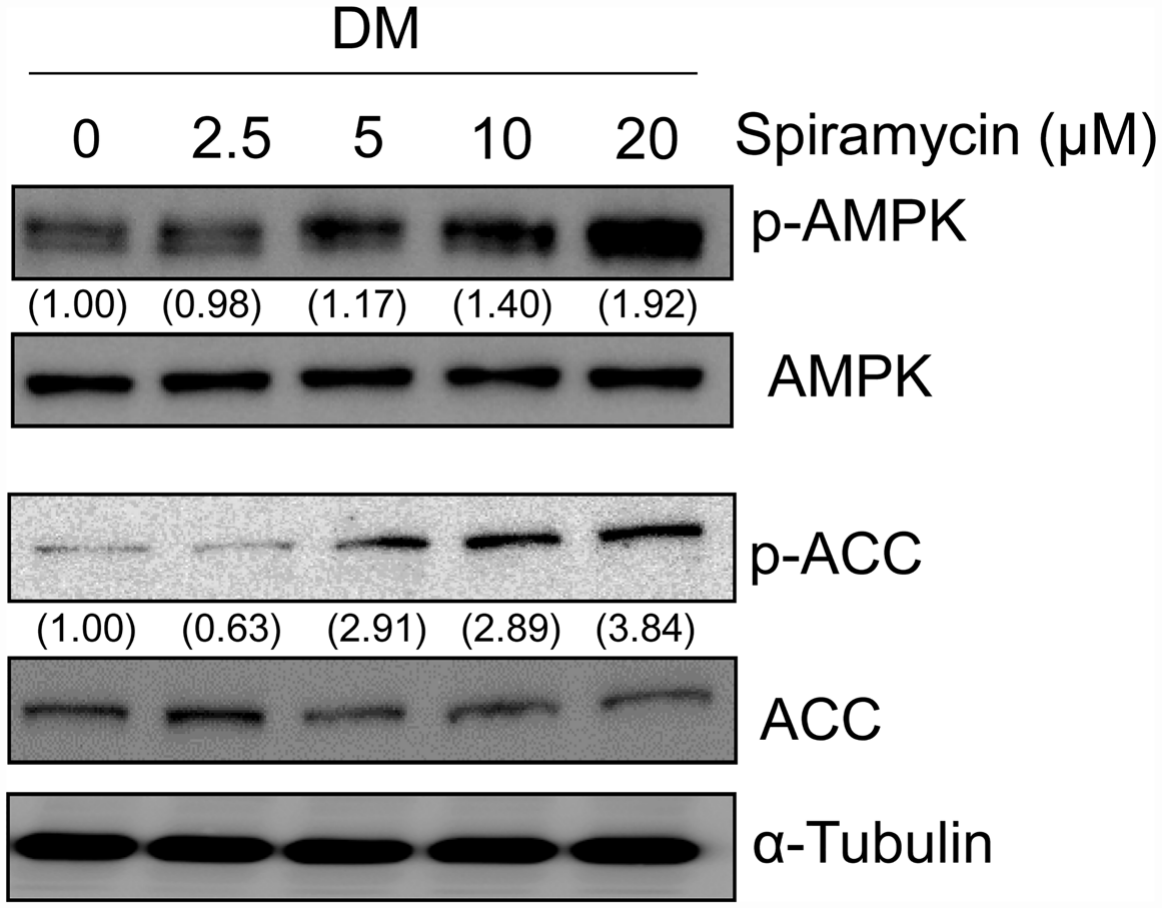
Effect of spiramycin on phosphorylation of AMPK and ACC during 3T3-L1 adipocyte differentiation. Confluent 3T3-L1 preadipocytes (Day 0) were incubated into DM including MDI with specified concentrations of spiramycin. After 1 h, protein levels of phosphorylated AMPK and ACC (p-AMPK and p-ACC) were analyzed by western blotting. The numbers at the bottom of the figure indicate the relative band intensity normalized to that of the non-phosphorylated protein (fold-change in comparison with that of the control group).

To determine the effect of spiramycin on AMPK activation, 3T3-L1 preadipocytes were treated with DM including DMI in the presence of spiramycin for 1h, and then the phosphorylation levels of AMPK and its substrate ACC was measured by western blotting. Interestingly, spiramycin at 20 μM increased phosphorylation of AMPK and ACC by about 1.9 and 3.8-fold, respectively (Fig 3). The effects of spiramycin on phosphorylation of AMPK and ACC were dose-dependent. Notably, AMPK activation by spiramycin at 20 μM was comparable with 2 mM of AICAR, a known AMPK agonist (S2 Fig).

To investigate whether the spiramycin directly binds to AMPK, *in silico* molecular docking simulations were performed. The docking results showed that the spiramycin can dock the AMP binding site in AMPK alpha domain (kinase) with higher docking energy than AICAR (a known activator) and that it also binds to AMPK gamma domain. As a negative control, metformin showed the highest binding energy out of three compounds (S3 Fig). These results suggest that spiramycin enhances AMPK activity in adipocytes, which may lead to inhibition of adipogenesis and energy storage.

### Spiramycin ameliorates HFD-induced obesity and obesity-related parameters in C57BL/6 mice

**Fig 4:**
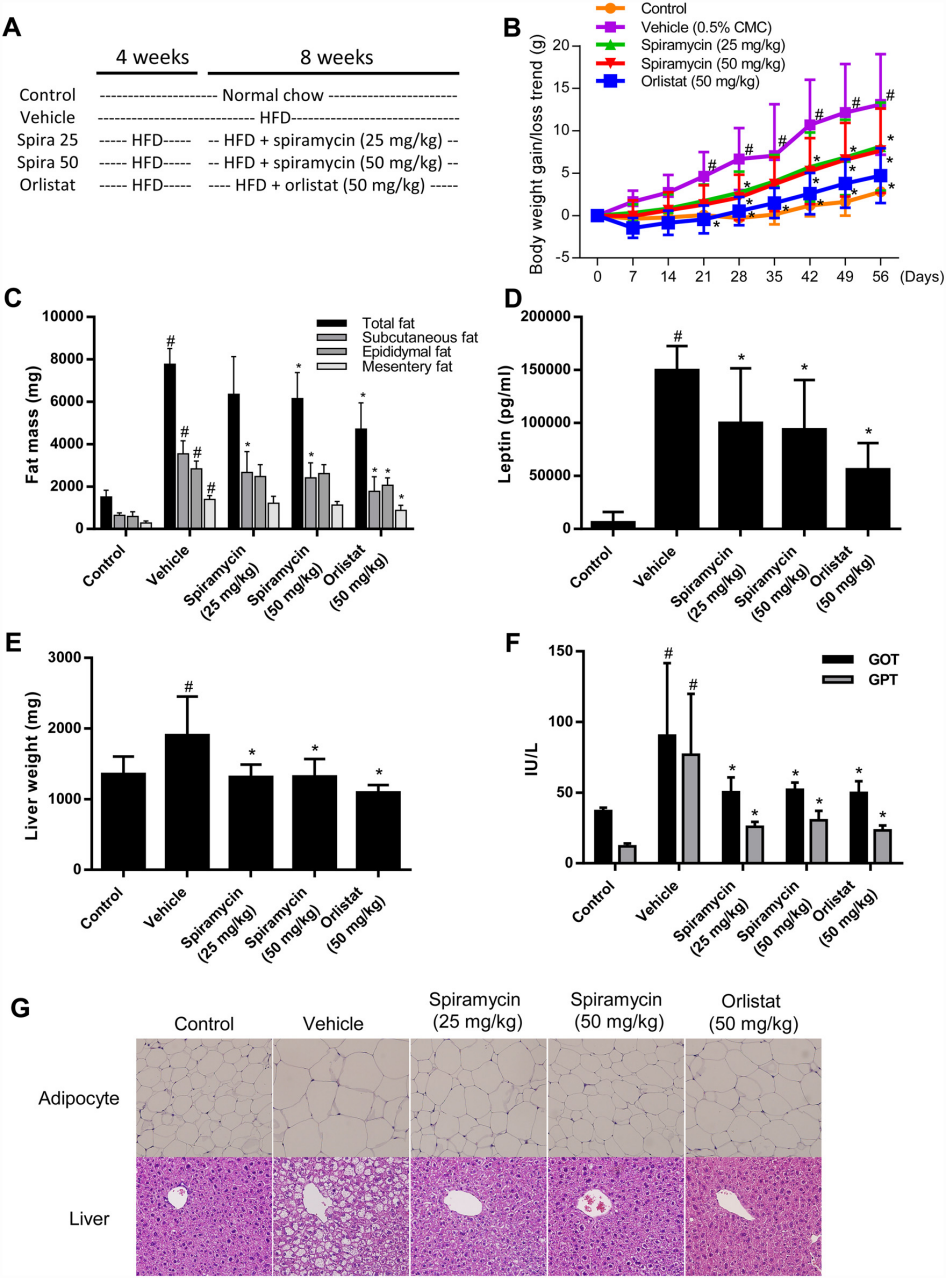
Effects of spiramycin on HFD-induced obesity in C57BL/6 mice. Spiramycin (25 or 50 mg/kg) or orlistat (50 mg/kg) were administered by oral gavage for 8 weeks while the mice were fed the HFD. (A) Experimental outline. (B) Body weight was measured 3 times per week. The group that received HFD alone(■) showed steady body weight gain, while the spiramycin-(▲ or ▼) and orlistat-treated(◆) groups showed significantly attenuated body weight gain. (C) Adipose tissue weight in subcutaneous, epididymal, and mesentery fat. (D) Amount of leptin in plasma measured by ELISA. (E) Liver weight. (F) Plasma levels of GOT and GPT measured using a chemical analyzer. (G) H&E-stained images of liver and subcutaneous adipose tissue samples from the normal diet (control), vehicle-treated, spiramycin-treated, and orlistat-treated groups. The results are expressed as the mean ± SD for each group (n = 8).

After feeding a HFD to C57BL/6 mice for 4 weeks, the body weight of the HFD group was significantly increased in comparison with that of the normal chow diet-fed control group. HFD-fed animals were divided into 4 groups: HFD alone (vehicle), HFD + spiramycin (25 or 50 mg/kg), and HFD + orlistat (50 mg/kg), which were administered for further 8 weeks (Fig 4A). Orlistat (also known as tetrahydrolipstatin), an anti-obesity drug, served as a positive control [18]. Mice receiving 25 mg/kg or 50 mg/kg of spiramycin showed significantly reduced body weight gain during the spiramycin intervention period, reporting a mean gain of 9.4 g (P <0.05) and 9.0 g (P <0.05), respectively. Contrary to this, the HFD vehicle control group gained a mean of 15.1 g (Fig 4B and Table 2). The spiramycin-treated groups and the group that received the HFD alone showed similar daily food intake (Table 2). Any adverse effect including diarrhea was not observed in spiramycin-treated mice.

**Table 2:** The changes of body and organ weight, and food intake.

		+ HFD			
	Normal	Vehicle	Spiramycin25 mg/kg	Spiramycin 50 mg/kg	Orlistat 50 mg/kg
Initial weight (g)	26.5±0.7	30.5±1.4	30.4±1.4	30.5±1.8	30.4±1.7
Final weight (g)	29.3±1.3	45.6±3.7	39.8±5.3	39.5±4.9	35.9±3.6
Weight gain (g)	2.8±0.7	15.1±2.4#	9.4±4.0*	9.0±3.2*	5.5±2.0*
Total fat (g)	1.5±0.3	7.8±0.7#	6.3±1.8	6.1±1.2*	4.7±1.2*
Subcutaneous fat (g)	0.6±0.1	3.5±0.6#	2.7±1.0*	2.4±0.7*	1.8±0.7*
Epididymal fat (g)	0.6±0.2	2.8±0.4#	2.5±0.6	2.6±0.4	2.1±0.4*
Mesentary fat (g)	0.3±0.1	1.4±0.2#	1.2±0.3	1.1±0.2	0.9±0.3*
Liver (g)	1.4±0.2	1.9±0.5#	1.3±0.2*	1.3±0.2*	1.1±0.1*
Food intake (g/day)	3.9±0.6	3.7±0.8	3.3±0.8	3.3±0.8	3.8±0.9

Values are expressed as the mean ± SD (n = 8 per group). Statistical significance compared with the HFD group (One-way ANOVA followed by Dunnett’s multiple comparison test): *P<0.05. Statistical significance compared with the control group (One-way ANOVA followed by Dunnett’s multiple comparison test): #P<0.05.

Administration of spiramycin at 25 mg/kg reduced the relative subcutaneous fat mass (Fig 4C and Table 2) of treated mice in comparison with that of the group that received the HFD alone. However, spiramycin at 50 mg/kg significantly decreased total fat and subcutaneous fat mass in treated animals in comparison with those of the group that received the HFD alone (20.9% reduction in total fat and 32.0% reduction in subcutaneous fat mass). In accordance with these results, adipocyte size analysis in subcutaneous adipose tissue by H&E staining revealed that the size of the enlarged adipocytes in the HFD-fed mice was considerably reduced in the spiramycin-treated mice in comparison with that of the normal chow-fed control mice (Fig 4G, upper panels). Serum profiles were also analyzed (Table 3). The total cholesterol serum level of the group that received the HFD alone was significantly higher than that of the normal food-fed control group, while the spiramycin-administered HFD-mice showed significantly reduced total cholesterol and LDL-cholesterol levels in the serum, indicating improved lipid homeostasis. Serum HDL levels did not differ significantly among the group that received the HFD alone, the spiramycin-treated groups, and the orlistat-treated group. Interestingly, the triglyceride level of the group that received spiramycin at 50 mg/kg was slightly reduced in comparison with that of the group that received the HFD alone (which was marginally increased in comparison with that of the normal food-fed control group), although the difference was not statistically significant.

**Table 3:** Effects of spiramycin on GOT, GPT, and serum lipid levels in mice fed different experimental diets for 8 weeks.

		+ HFD			
	Normal	Vehicle	Spiramycin25 mg/kg	Spiramycin 50 mg/kg	Orlistat 50 mg/kg
Triglyceride (mg/dl)	68.0±43.3	93.0±40.7	80.3±26.1	66.9±18.7	40.9±17.1*
Total Cholesterol (mg/dl)	119.0±15.9	211.6±31.5#	182.5±20.9*	190.0±21.9	178.6±15.4*
HDL-C (mg/dl)	74.0±10.5	104.3±13.0#	105.3±10.3	110.1±5.8	101.5±5.5
LDL-C (mg/dl)	6.0±0.8	9.3±2.4#	6.4±0.9*	6.5±1.8*	6.6±0.9*
Total cholesterol/ HDL	1.6±0.7	2.0±0.9#	1.7±0.7*	1.7±0.7*	1.8±0.7*
GOT (IU/L)	36.9±2.5	90.4±51.2#	50.3±10.4*	52.1±5.0*	49.8±8.3*
GPT (IU/L)	12.0±2.1	76.8±43.1#	25.9±3.4*	30.5±6.6*	23.3±3.5*

Values are expressed as the mean ± SD (n = 8 per group). Statistical significance compared with the HFD group (One-way ANOVA followed by Dunnett’s multiple comparison test): *P<0.05. Statistical significance compared with the control group (One-way ANOVA followed by Dunnett’s multiple comparison test): #P<0.05. GOT, glutamate oxaloacetate transaminase; GPT, glutamate pyruvate transamiase.

Besides hypertriglyceridemia, hypercholesterolemia, and increased cholesterol in LDL and VLDL fractions, reduced cholesterol in HDL is also an important feature for a pro-atherogenic lipid profile. Thus, we calculated the ratio of cholesterol to HDL for each groups. The ratio in HFD mice (2.0) was higher than the control group (1.6), while HFD mice treated with 25 mg/kg, 50 mg/kg of spiramycin, or 50 mg/kg of orlistat showed the reduced ratio values, 1.7, 1.7, and 1.8, respectively.

Leptin production is closely associated with adiposity [19]; therefore, we measured serum leptin levels in mice. The serum leptin level of mice fed the HFD alone was significantly increased by approximately 24-fold in comparison with that of the normal control group. However, 25 and 50 mg/kg of spiramycin significantly attenuated the HFD-induced increase in serum leptin level by approximately 33% and 37%, respectively (Fig 4D).

A HFD can lead to hepatic steatosis, inflammation, and injury, which can subsequently lead to nonalcoholic fatty liver disease (NAFLD) and steatohepatitis [20]. After 12 weeks of HFD, the liver weight and serum GOP and GTP levels of the mice that received the HFD alone were significantly increased in comparison with those of the control group. These results indicated that hepatic steatosis and injury occurred in the group that received the HFD alone (Fig 4E and 4F; Table 3). Consistent with this finding, histological examination of the group that received HFD alone via H&E staining showed significant lipid droplet accumulation in the liver (Fig 4G, lower panels). However, administration of spiramycin at 25 and 50 mg/kg markedly attenuated the HFD-induced increases in liver weight, GOT and GTP levels, and hepatic lipid droplet formation in a dose-dependent manner. These results strongly suggest that spiramycin ameliorates hepatic steatosis and injury in HFD-fed mice. The *in vivo* efficacy of spiramycin was slightly less than that of FDA approved anti-obesity drug orlistat at the same concentrations.

## Discussion

Spiramycin, a macrolide antibiotic, is highly active against Gram-positive bacteria and mycoplasma species and is widely used to treat toxoplasmosis and other soft-tissue infections in cattle, swine, poultry, and sheep [21]. In the course of our search for molecules showing anti-adipogenic activity, we identified spiramycin as a potential hit compound. Here, for the first time, we show the molecular mechanism underlying the anti-adipogenic effects of spiramycin in 3T3-L1 cells and the *in vivo* effect of spiramycin in HFD-induced obese mice.

Our results show that spiramycin significantly inhibits preadipocyte differentiation by attenuating MDI-induced lipid accumulation (Fig 1). Master transcriptional regulators of adipogenesis, including PPARγ, C/EBPα, and SREBP1c, are necessary modulators of target gene expression involved in adipogenesis and differentiation at various stages [7, 8]. Interestingly, spiramycin decreases expression of PPARγ, C/EBPα, and SREBP1 at the transcriptional and translational levels, thereby reducing expression of their target genes, including FAS, aP2, and GLUT4 (Fig 2). FAS is an enzyme that catalyzes the synthesis of long-chain fatty acids from acetyl-CoA and malonyl-CoA in 3T3-L1 adipocytes. Another adipocyte marker, aP2, is a carrier protein for fatty acids and an important facilitator of lipid transport, uptake, and metabolism [22]. Therefore, our results suggest that spiramycin inhibits *de novo* triglyceride synthesis and adipocyte differentiation.

AMPK is a heterotrimeric serine/threonine protein kinase that is widely expressed in various tissues, including the liver, adipose tissue, brain, and skeletal muscle [23]. AMPK is a master metabolic regulator responsible for modulating cellular metabolism. AMPK regulates lipid and glucose metabolism by directly phosphorylating enzymes and regulatory proteins. AMPK is also regulates adipocyte differentiation by modulating adipogenic transcription factors and fatty acid synthesis in 3T3-L1 adipocytes. Thus, AMPK can be a promising target for treating metabolic disorders such as obesity and type 2 diabetes [24]. Emerging evidence suggests that natural AMPK activator compounds such as sulforaphane [25], ursolic acid [26], and AICAR (an analog of adenosine monophosphate (AMP)) [27] inhibit adipogenesis by activating AMPK. AICAR-induced AMPK activation blocks adipocyte differentiation by down-regulating several adipocyte-specific transcription factors [28]. AICAR also effectively restores metabolic alterations in mice with HFD-induced obesity [27]. In addition, increased lipid accumulation in the liver of HFD-fed obese rats with reduced hepatic AMPK phosphorylation is ameliorated by AMPK activation [29, 30]. These findings indicate that activation of AMPK inhibits adipogenesis in preadipocytes and ameliorates obesity and obesity-associated hepatic steatosis.

Our results revealed that spiramycin induces AMPK activation more effectively, compared with AICAR in 3T3-L1 cells during early adipogenesis (S2 Fig), thereby inhibiting the activity of master transcriptional regulators of adipogenesis. Furthermore, we showed that AMPK activation by spiramycin increased ACC phosphorylation at Ser79 (Fig 3A). Usually, inactivated ACC by phosphorylation, resulting in a decrease in malonyl-CoA synthesis, leading to increased β-oxidation and triglyceride consumption during energy production [31]. Consistent with these, *in silico* docking simulations show that spiramycin might bind at the AMP binding site in AMPK gamma domain like as AICAR (known as an AMP mimic) (S3 Fig). Therefore, our results strongly suggest that AMPK activation by spiramycin causes ACC inactivation, facilitating fatty acid oxidation and reducing lipid accumulation in 3T3-L1 adipocytes.

Our study presents several lines of *in vivo* evidence demonstrating that spiramycin has anti-obesity effects in HFD-fed mice. First, spiramycin reduced body weight gain. Second, serum leptin levels, which reflect adipose tissue growth in obese animals, were significantly reduced by spiramycin treatment, suggesting that spiramycin improved leptin resistance in HFD-fed mice. Third, spiramycin-treated HFD-fed mice lost adipose tissue mass. Finally, spiramycin reduced hepatic lipid accumulation in HFD-fed obese mice. Moreover, decreased serum levels of GPT and GOT suggested that spiramycin attenuated the hepatic injury caused by HFD. Taken together, our *in vivo* results in mice provide solid evidence for the anti-obesity effect of spiramycin. Further studies are required to address whether spiramycin directly binds to AMPK like AICAR or to upstream proteins such as LKB1, CAMKK2, or insulin receptors. It may also be possible that spiramycin inhibits C/EBP activation or expression, perhaps by negatively regulating MAPK/glycogen synthase kinase 3β, PKA, or JAK/STAT signaling [2]. To use a remedy for obesity in human, spiramycin could be combined with functional foods or nutraceutical products such as Sango sprout juice [32] to further improve its anti-obesity effect in a safer way.

## Conclusion

Spiramycin ameliorates HFD-induced obesity and associated hepatic steatosis by inhibiting adipogenesis. Our findings provide a basis for the future development of novel therapeutic strategies for obesity management.

## Supporting Information

S1 Fig Schematic representation of process for adipocyte differentiation and cell viability of spiramycin. (A) A schematic diagram showing adipocyte differentiation procedure from post-confluent 3T3-L1 cells (day 0) by replacing differentiation medium (DM). Details are as described in “Materials and Methods”. On day 6 after differentiation, Oil-red O staining, TG assay, qRT-PCR and western blot were performed. (B) Cell viability was assessed by CCK-8 assay kit. Values are presented as OD at 450 nm (mean ± SEM, n = 3).
(DOCX)

S2 Fig Activation of AMPK by Spiramycin or AICAR in 3T3-L1 preadipocytes. Confluent 3T3-L1 cells (day 0) were treated with DM including MDI in the presence of spiramycin or AICAR for 1 hr. Cell lysates were then analyzed by western blotting for phosphorylated and total AMPK, phosphorylated and total ACC. AICAR (2 mM) was used as a positive control for AMPK activation. The numbers at the bottom of the figure indicate the relative band intensity normalized to that of the non-phosphorylated protein (fold-change in comparison with that of the control group).
(DOCX)

S3 Fig Molecular docking results. (A) AMP binding site predictions in AMPK gamma domain. Yellow spheres in the molecular models represent the AMP binding regions. (Heat map) Analogues of AMP, such as ATP, NAI, ADP (compounds are named in PDB) have been also found as possible candidates for the AMP binding site. (B) Docking results of gamma domain with three compounds: spiramycin, metformin, and AICAR. The docking energy scores are below structures (unit is kcal/mol). AICAR and spiramycin are predicted as a agonist to AMP binding sites. A protein structure is drawn by white cartoon image and the AMP binding sites are shaded by red. The compound are drawn by blue wireframe model.
(DOCX)
