# Supplementary material for: Anti-Obesity Effects of Spiramycin In Vitro and In Vivo
Source: PLoS One. 2016 Jul 11;11(7):e0158632. doi: 10.1371/journal.pone.0158632 (PMC4939947; doi:10.1371/journal.pone.0158632)
Supplement: S1 Fig — (A) A schematic diagram showing adipocyte differentiation procedure from post-confluent 3T3-L1 cells (day 0) by replacing differentiation medium (DM). Details are as described in “Materials and Methods”. On day 6 after differentiation, Oil-red O staining, TG assay, qRT-PCR and western blot were performed. (B) Cell viability was assessed by CCK-8 assay kit. Values are presented as OD at 450 nm (mean ± SEM, n = 3). (DOCX) [file pone.0158632.s001.docx]

**Supplementary Information**

**Anti-obesity effects of spiramycin *in vitro* and *in vivo***

**Mun Ock Kim *et al.***

**● Supplementary Method**

**● Supplementary Figure**

**- S1 Fig.** Schematic representation of process for adipocyte differentiation and

cell viability of spiramycin.

**Supplemental Method**

**Cell viability assay**

Cell viability was assessed using the cell counting kit (CCK-8; Dojindo Lab, Kumamoto, Japan) according to the manufacturer’s instructions. Briefly, cells were seeded onto 96-well plates at a density of 8 × 10^3^ cells/well. After 24 hr, the cells were stimulated with DM including MDI with exposure to spiramycin for 2 days. The medium was replaced with DM including insulin only without IBMX and dexamethasone. After 2 days, the cells were re-fed with fresh high-glucose DMEM including 10% FBS. On day 6 after the differentiation (day 6, Supplemental Figure 1A), 10 μL of reaction solution was added to the cultured cells in 100 μL culture medium, followed by incubation at 37 °C for 1 hr. The absorbance was measured at wavelength of 450 nm using a VERSA max microplate reader (Molecular Devices, Sunnyvale, CA, USA) and the measured absorbance was converted to the percentage (%) of the control value.


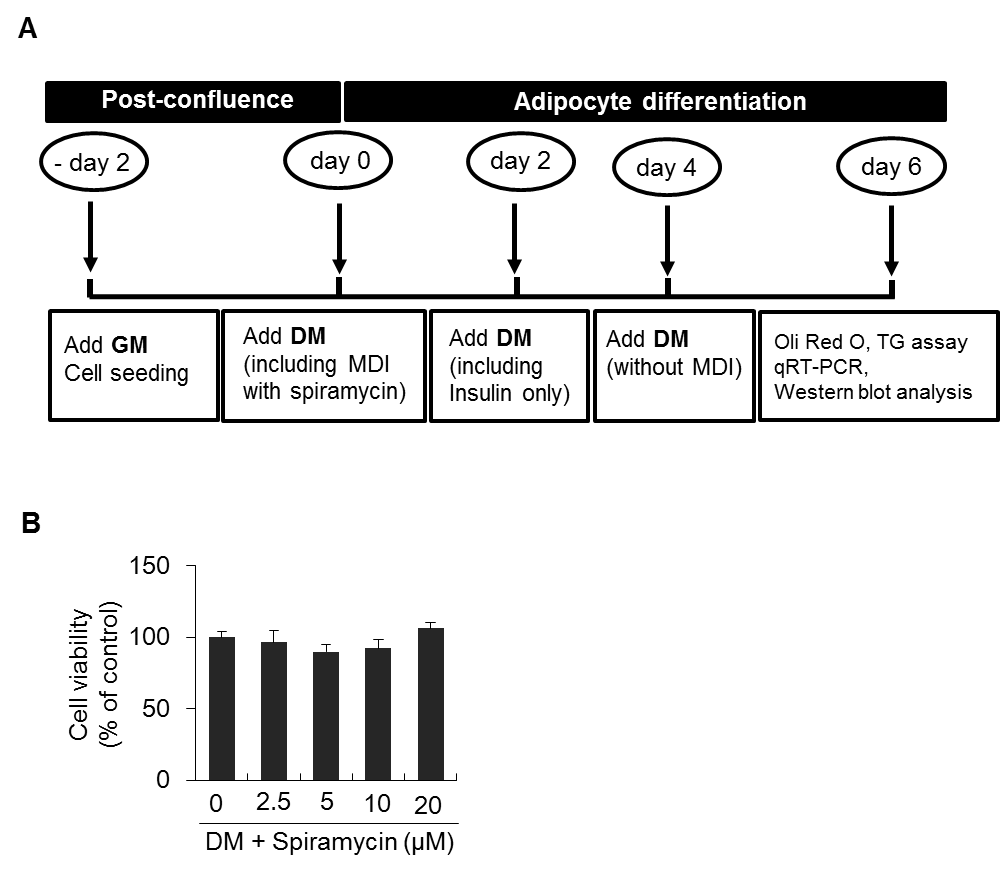


**S1 Fig.** Schematic representation of process for adipocyte differentiation and cell viability of spiramycin. (A) A schematic diagram showing adipocyte differentiation procedure from post-confluent 3T3-L1 cells (day 0) by replacing differentiation medium (DM). Details are as described in “Materials and Methods”. On day 6 after differentiation, Oil-red O staining, TG assay, qRT-PCR and western blot were performed. (B) Cell viability was assessed by CCK-8 assay kit. Values are presented as OD at 450 nm (mean ± SEM, n=3).
