# Supplementary material for: Anti-Obesity Effects of Spiramycin In Vitro and In Vivo
Source: PLoS One. 2016 Jul 11;11(7):e0158632. doi: 10.1371/journal.pone.0158632 (PMC4939947; doi:10.1371/journal.pone.0158632)
Supplement: S2 Fig — Confluent 3T3-L1 cells (day 0) were treated with DM including MDI in the presence of spiramycin or AICAR for 1 hr. Cell lysates were then analyzed by western blotting for phosphorylated and total AMPK, phosphorylated and total ACC. AICAR (2 mM) was used as a positive control for AMPK activation. The numbers at the bottom of the figure indicate the relative band intensity normalized to that of the non-phosphorylated protein (fold-change in comparison with that of the control group). (DOCX) [file pone.0158632.s002.docx]

**Supplementary Information**

**Anti-obesity effects of spiramycin *in vitro* and *in vivo***

**Mun Ock Kim *et al.***

**
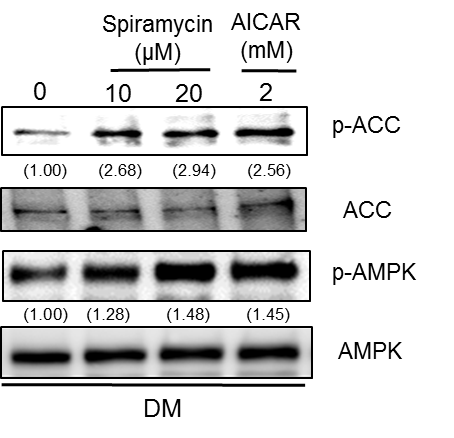
**

**S2 Fig.** Activation of AMPK by Spiramycin or AICAR in 3T3-L1 preadipocytes. Confluent 3T3-L1 cells (day 0) were treated with DM including MDI in the presence of spiramycin or AICAR for 1 hr. Cell lysates were then analyzed by western blotting for phosphorylated and total AMPK, phosphorylated and total ACC. AICAR (2 mM) was used as a positive control for AMPK activation. The numbers at the bottom of the figure indicate the relative band intensity normalized to that of the non-phosphorylated protein (fold-change in comparison with that of the control group).
