# Supplementary material for: Anti-Obesity Effects of Spiramycin In Vitro and In Vivo
Source: PLoS One. 2016 Jul 11;11(7):e0158632. doi: 10.1371/journal.pone.0158632 (PMC4939947; doi:10.1371/journal.pone.0158632)
Supplement: S3 Fig — (A) AMP binding site predictions in AMPK gamma domain. Yellow spheres in the molecular models represent the AMP binding regions. (Heat map) Analogues of AMP, such as ATP, NAI, ADP (compounds are named in PDB) have been also found as possible candidates for the AMP binding site. (B) Docking results of gamma domain with three compounds: spiramycin, metformin, and AICAR. The docking energy scores are below structures (unit is kcal/mol). AICAR and spiramycin are predicted as a agonist to AMP binding sites. A protein structure is drawn by white cartoon image and the AMP binding sites are shaded by red. The compound are drawn by blue wireframe model. (DOCX) [file pone.0158632.s003.docx]

**Supplementary Information**

**Anti-obesity effects of spiramycin *in vitro* and *in vivo***

**Mun Ock Kim *et al.***

**● Supplementary Method**

**● Supplementary Figure**

**- S3 Fig.** Molecular docking results.

**Supplemental Method**

**Protein-ligand docking simulations**

AMPK protein was revealed in PDB (Protein Data Bank: PDB ID: 4CFE). It consists of three domain structures, alpha (protein kinase), beta (glycogen binding site), and gamma (AMP-activated protein kinase subunit). We extracted them individually and ran three docking simulations with three compounds: spiramycin, metformin (non-direct target of AMPK), and AICAR (known direct target of AMPK as an AMP analog). Ligand binding sites in those structures were predicted (Supplemental Figure 3). Gamma structures has common AMP binding regions (beta structure has no AMP binding regions). As a negative control, metformin shows the highest binding energy, compared with other two compounds.

| 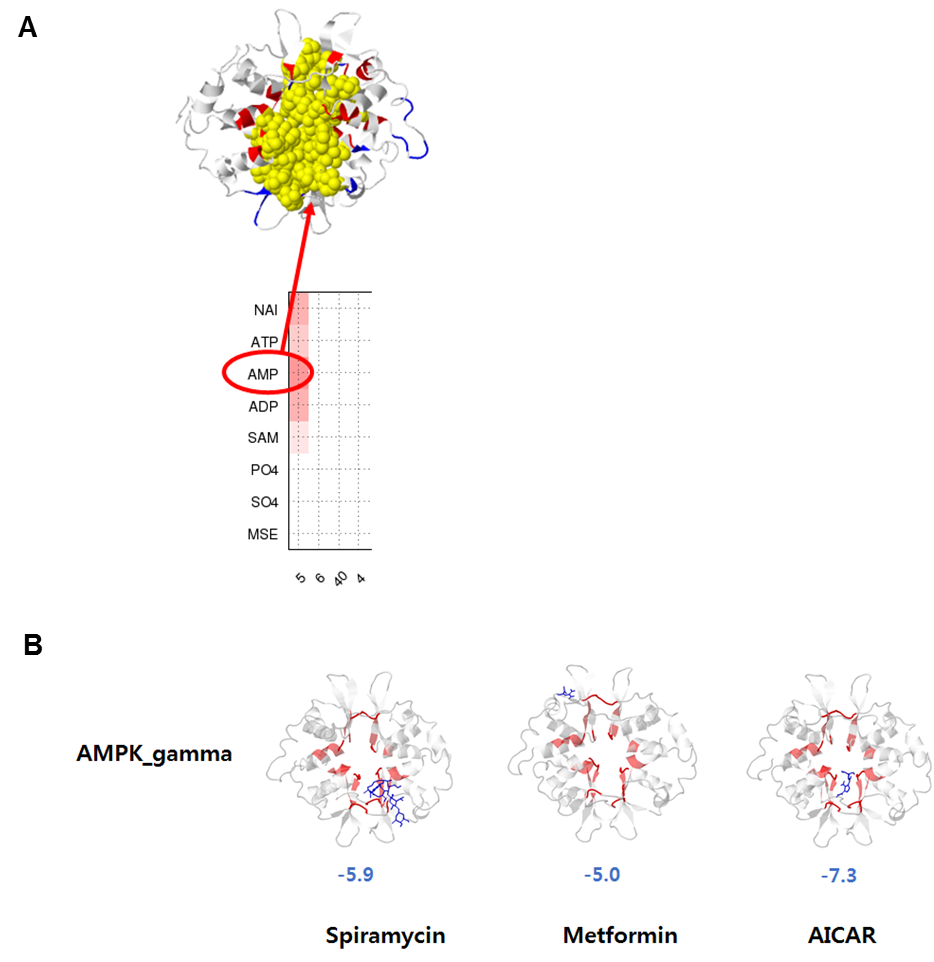 |
| --- |
|  |
| **S3 Fig.** Molecular docking results. (A) AMP binding site predictions in AMPK gamma domain. Yellow spheres in the molecular models represent the AMP binding regions. (Heat map) Analogues of AMP, such as ATP, NAI, ADP (compounds are named in PDB) have been also found as possible candidates for the AMP binding site. (B) Docking results of gamma domain with three compounds: spiramycin, metformin, and AICAR. The docking energy scores are below structures (unit is kcal/mol). AICAR and spiramycin are predicted as a agonist to AMP binding sites. A protein structure is drawn by white cartoon image and the AMP binding sites are shaded by red. The compound are drawn by blue wireframe model. |
